# The careful control of Polo kinase by APC/C-Ube2C ensures the intercellular transport of germline centrosomes during *Drosophila* oogenesis

**DOI:** 10.1098/rsob.200371

**Published:** 2021-06-30

**Authors:** Alexis Leah Braun, Francesco Meghini, Gema Villa-Fombuena, Morgane Guermont, Elisa Fernandez-Martinez, Zhang Qian, Maria Dolores Martín-Bermudo, Acaimo González-Reyes, David Moore Glover, Yuu Kimata

**Affiliations:** ^1^ Department of Genetics, University of Cambridge, Cambridge CB2 3EH, UK; ^2^ Centro Andaluz de Biología del Desarrollo, CSIC/Universidad Pablo de Olavide/JA, Carretera de Utrera km 1, 41013 Sevilla, Spain; ^3^ School of Life Science and Technology, ShanghaiTech University, 393 Middle Huaxia Road, Shanghai 201210, People's Republic of China

**Keywords:** *Drosophila*, germline, centrosomes, Polo kinase, APC/C, fertility

## Abstract

A feature of metazoan reproduction is the elimination of maternal centrosomes from the oocyte. In animals that form syncytial cysts during oogenesis, including *Drosophila* and human, all centrosomes within the cyst migrate to the oocyte where they are subsequently degenerated. The importance and the underlying mechanism of this event remain unclear. Here, we show that, during early *Drosophila* oogenesis, control of the Anaphase Promoting Complex/Cyclosome (APC/C), the ubiquitin ligase complex essential for cell cycle control, ensures proper transport of centrosomes into the oocyte through the regulation of Polo/Plk1 kinase, a critical regulator of the integrity and activity of the centrosome. We show that novel mutations in the APC/C-specific E2, Vihar/Ube2c, that affect its inhibitory regulation on APC/C cause precocious Polo degradation and impedes centrosome transport, through destabilization of centrosomes. The failure of centrosome migration correlates with weakened microtubule polarization in the cyst and allows ectopic microtubule nucleation in nurse cells, leading to the loss of oocyte identity. These results suggest a role for centrosome migration in oocyte fate maintenance through the concentration and confinement of microtubule nucleation activity into the oocyte. Considering the conserved roles of APC/C and Polo throughout the animal kingdom, our findings may be translated into other animals.

## Graphical abstract

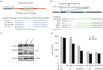

## 1. Introduction

The centrosome is a major microtubule-organizing centre (MTOC) in many animal cells and, through microtubule nucleation, regulates a wide range of cellular processes during development and in adult tissues [1,2]. The centrosome is composed of a pair of centrioles and an amorphous proteinaceous matrix, the pericentriolar material (PCM), which recruits microtubule-nucleating proteins such as γ-tubulins and also contributes to the stability of centrioles [3,4]. In dividing cells, the centrosomes facilitate the assembly and the orientation of the mitotic spindle, thereby ensuring accurate chromosome segregation and impacting cell fate decision and tissue architecture. In non-dividing cells, centrosomes direct cell migration and intracellular transport by forming the polarized microtubule network, or alternatively centrioles are converted to basal bodies to form primary cilia. Centrosome dysfunction is linked to various human diseases including cancer, microcephaly and ciliopathy [1,5].

In the fruit fly *Drosophila melanogaster*, the centrosome has been implicated in the development of the oocyte through their microtubule-organizing capabilities [6,7]. The *Drosophila* oocyte develops through 14 stages that are delineated by morphological changes of egg chambers [8,9]. In each ovariole, oogenesis starts at the anterior tip in the germarium where the female germline stem cell resides and divides asymmetrically to produce a daughter stem cell and a cystoblast. The cystoblast then undergoes exactly four rounds of mitotic divisions with incomplete cytokinesis to create a 16-cell syncytial cyst, in which only one cell eventually becomes the oocyte, whereas the other 15 cells take on a nurse cell fate. During this process, germline centrosomes of the 16 cystocytes exhibit a very peculiar behaviour: virtually, all the 32 centrosomes intercellularly migrate within the cyst to eventually accumulate in one cell, the future oocyte [7]. This migration coincides with the events that mark the specification of the oocyte: the concentration of fate determinants such as Bicaudal-D (BicD), Egalitarian (Egl) and Oo18 RNA-binding protein (Orb) [10–13], and the restriction of synaptonemal complexes to the oocyte [14]. These observations suggest potential involvement of germline centrosomes in the specification and/or the subsequent differentiation of the oocyte [6]. Importantly, the centrosome migration following the syncytial cyst formation is likely to be a universal feature of oogenesis as it is observed in animals as diverse as fish and mammals [15,16]. However, despite the universality of this phenomenon, its significance, as well as the molecular mechanism directing this process, remains elusive.

The microtubule cytoskeleton plays a critical role in *Drosophila* oogenesis [17,18]. Shortly after the formation of the 16-cell cyst, microtubules start nucleating in the presumptive oocyte and subsequently form an oocyte-centric polarized network that runs through the cyst, which is believed to be required for oocyte maturation as well as the subsequent establishment of the body axes of the embryo [9,19,20]. Given the established role of the centrosome as a major MTOC, it has been postulated that centrosomes may be involved in the formation and/or the maintenance of this oocyte-centred microtubule network through their microtubule nucleation activity [6,17,21]. Nevertheless, the study using loss-of-function mutations in *sas4*, a gene required for centriole duplication, demonstrated that the oocyte develops normally in homozygous *sas4* mutant flies, despite virtually no detectable centrioles in the female germlines [22]. Moreover, it is widely acknowledged that germline centrosomes are degenerated by the end of oogenesis and do not contribute to the embryo. It was recently demonstrated in *Drosophila* that forced retention of centrosomes in the oocyte results in meiotic defects and subsequent sterility [23]. Thus, there is a clear paradox: if the centrosomes are unnecessary for oocyte development, and even detrimental to embryogenesis, why do they need to be transported into the oocyte? Why is such a seemingly wasteful phenomenon conserved through evolution?

In our previous study, we showed that the centrosome migration requires neither microtubule polymerization nor the activity of BicD and Egl, but does the function of the minus end-directed microtubule motor dynein [24]. It was also reported that mutations in the large cytoskeletal linker, spectroplakin family protein, Short stop (Shot), block the centrosome migration [25]. However, the function of dyneins is also required for the normal morphology of the fusome, the germline-specific membranous organelle that forms a large branched network throughout the cyst and plays a critical role in the asymmetric division of cystoblasts as well as in the specification of the oocyte [21,24,26]. In the *shot* mutants, although the fusome structure appears unaffected, the polarized microtubule network is not formed and the fate markers do not accumulate in the oocyte [25]. Thus, the interdependency between the events that coincide upon oocyte specification has been a stumbling block for assigning a specific role for the centrosome migration during *Drosophila* oogenesis.

Both the biogenesis and the microtubule nucleation activity of the centrosome are tightly coupled to the progression of the cell cycle. An E3 ubiquitin ligase complex, Anaphase Promoting Complex/Cyclosome (APC/C), plays a central role in this coupling. Besides many proteins involved in cell cycle regulation, the APC/C also targets proteins that are critical for centriole biogenesis, such as Sas6, STIL and Securin, and for microtubule nucleation, such as Plk1, Aurora A and Spd2, for proteasome-mediated destruction within specific time windows during the cell cycle [27–32]. Conversely, APC/C activity is also regulated by the centrosome: the local activity of APC/C at centrosomes depends on the physical interaction between an APC/C regulatory subunit and a centrosome component [31,33]. Ube2C is the E2 ubiquitin-conjugating enzyme that is specifically required for the E3 ubiquitin ligase activity of APC/C [34,35]. The cellular levels of Ube2C oscillate during the cell cycle through its autoubiquitination, peaking at mitosis and dropping in the G1 phase [34,36,37]. Importantly, in *Drosophila* and mammalian cells, Ube2C is enriched at the centrosome and its over-expression leads to the amplification of centrosomes [34,38]. Moreover, excess Ube2C increases sporadic tumour formation in mice and over-expression of Ube2C is associated with various human cancers [38,39]. Therefore, strict control of Ube2C activity is critical for the tight coupling of the centrosome cycle to the cell cycle as well as for tumour suppression. However, the target(s) of the APC/C^Ube2C^ that is critical for centrosome integrity is as yet unidentified.

In this study, we report novel mutants of *Drosophila* Ube2C Vihar (Vih), *vih*^Δ*N*^, generated by CRISPR genome editing, in which the centrosome migration is intervened in the female germline, despite apparent intact fusome structure and proper oocyte specification. We show that *vih*^Δ*N*^ mutations probably cause the upregulation of APC/C, which in turn leads to the reduction of Polo kinase, one of the APC/C substrates and the critical regulator of the integrity and microtubule nucleation activity of the centrosome, in developing egg chambers. The reduction of Polo interferes with the transport of centrosomes into the oocyte, probably through destabilization of germline centrioles. We further show that, in the *vih*^Δ*N*^ mutant egg chambers with mislocalized centrosomes, the polarized microtubule network is weakened and microtubule nucleation occurs at ectopic centrosomes in nurse cells, resulting in the loss of oocyte fate and the disruption of the oocyte–nurse cell membranes. These results point to the critical importance of the centrosome transport for the spatial regulation of microtubule nucleation during the early oocyte development in *Drosophila*, which is orchestrated by the evolutionarily conserved pathway: APC/C^Vih^-Polo pathway.

## 2. Results

### 2.1. N-terminal deletion mutations in *Drosophila vih* gene cause defects in early oogenesis

**Figure 1.**
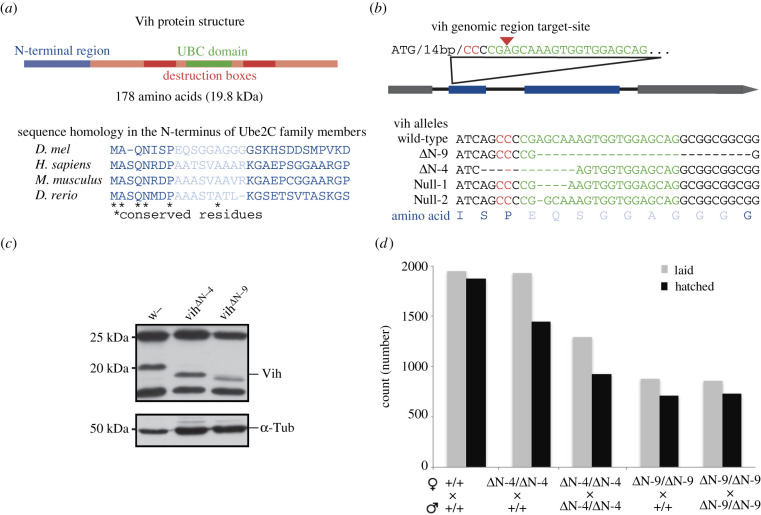
CRISPR/Cas9-generated *vih*^Δ*N*^ alleles. (*a*) Vih protein domains. Protein homology in vertebrates for the gRNA-targeted region, the residues deleted in the *vih*^Δ*N-9*^ allele are light blue. (*b*) CRISPR/Cas9 genomically engineered gRNA target-site design within the coding region of the gene. The red arrow denotes the cut site, the red residues denote the protospacer adjacent motif (PAM) sequence and the green residues denote the gRNA complementary sequence. Four examples of *vih* alleles that were created. (*c*) Western blot of protein extracts isolated from the ovaries of control and the two *vih*^Δ*N*^ alleles, blotted and stained with Vih and α-Tubulin (Tub) antibodies. (*d*) Eggs laid and larvae hatched for control, homozygous *vih*^Δ*N-4*^ or *vih*^Δ*N-9*^ females crossed to wild-type males, or to *vih*^Δ*N-4*^ or *vih*^Δ*N-9*^ males, were measured with 2 h samples collected every 1–2 days over a 14-day period.

To investigate the function of the APC/C-dependent regulation of centrosomes in animal development, we generated novel mutations in the APC/C-specific E2 enzyme, Vih, using the CRISPR genome editing technique in *Drosophila* [40]. We targeted the conserved 27-amino acid N-terminus extension of the Vih protein, which is unique to the Ube2C family E2s and is involved in auto-inhibition of the APC/C [37,41] (figure 1*a*). We have generated a series of *vih* alleles, of which two are null alleles (*vih^Null^*), caused by frame-shifts, and the other two are in-frame deletions which lack four or nine residues in the N-terminal extension (*vih^ΔN-4^* and *vih^ΔN-9^*; figure 1*b*; see Material and methods for detailed characterization). *vih^Null^* homozygous mutants were lethal at the pupal stage and showed various mitotic phenotypes, such as overly condensed chromosomes, lagging chromosomes and supernumerary centrosomes, in the larval neuroblasts, similar to the previously reported *vih* null alleles (electronic supplementary material, figure S1) [34]. By contrast, both *vih^ΔN^* mutants are homozygous viable and develop into adulthood. The endogenous expression of the truncated forms of Vih proteins was confirmed by western blot using the lysates of the *vih^ΔN^* mutant ovaries (figure 1*c*). *vih^ΔN^* adult flies appear morphologically normal, except for occasional minor defects. However, the females of both *vih^ΔN^* mutants showed a substantial decrease in cumulative fecundity (total number of eggs laid), down to 55% of control (figure 1*d*). Homozygous and heterozygous *vih^ΔN-9^* mutants showed comparable levels of fecundity reduction (figure 1*d*), suggesting a potential dominant effect of the mutation. In addition, while the majority of eggs laid by *vih^ΔN^* females could produce larva, a small proportion of eggs showed dorsal appendage defects (9%, *n* = 836, data not shown), indicative of dorsal–ventral polarity abnormalities, which are frequently caused by a defect in the microtubule-dependent patterning of the egg chamber [18]. These data suggest defects in oogenesis in *vih^ΔN^* mutant females.

**Figure 2.**
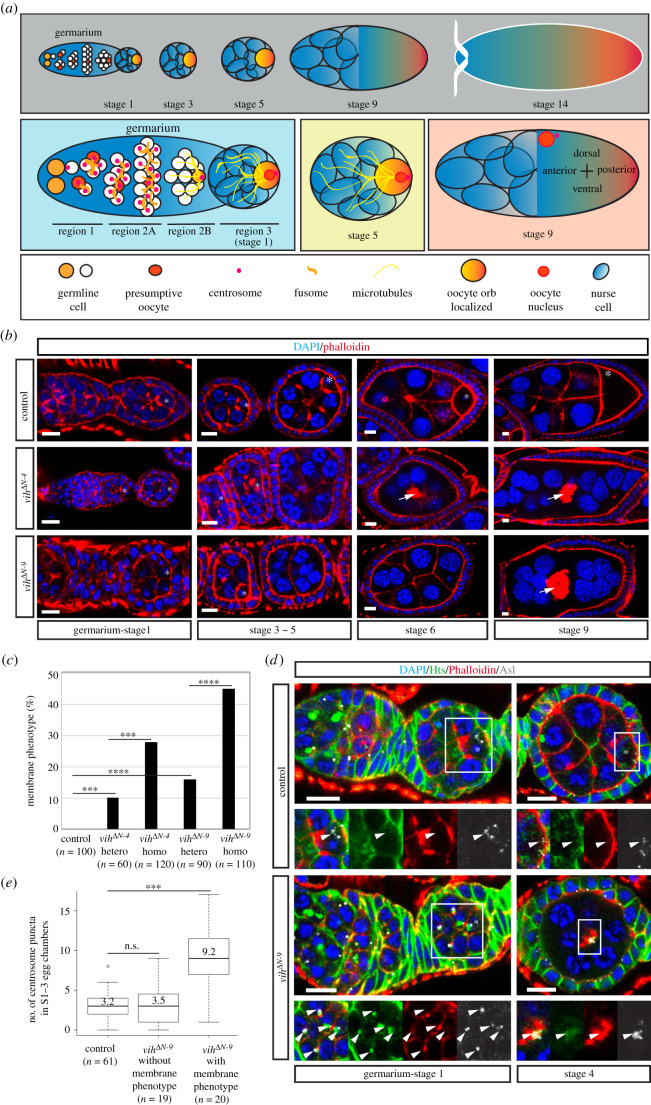
Egg chamber structure and centrosome transport is disrupted using the *vih*^Δ*N*^ alleles. (*a*) Schematic of egg chamber development. (*b*) Development time-series of phenotypic presentation for control, *vih*^Δ*N-4*^ and *vih*^Δ*N-9*^ egg chambers, stained for DNA (DAPI) in blue and F-actin (Phalloidin) in red. White arrows indicate actin aggregates and asterisks the oocytes. (*c*) The quantification of ovarioles that exhibited the membrane phenotype. The proportion of ovarioles with the membrane phenotype was determined for each of the genotypes indicated. (*d*) Germarium-S1 and S4 control and *vih*^Δ*N-9*^ egg chambers stained for DNA (DAPI) in blue, fusome (Hts) in green, and F-actin (Phalloidin) in red, and centrioles (Asl) in white (arrowheads indicate centrioles and asterisks the oocyte). (*e*) The quantification of centrosome puncta (stained with Asl) in S1–3 control and *vih*^Δ*N-9*^ egg chambers, significance determined using Welch's *t*-test for the control compared with the phenotype (*p* = 5.0 × 10^−6^) and with Wilcoxon signed-rank test with the control to no phenotype (*p* > 2 × 10^−1^). All scale bars equal to 10 µm.

To elucidate the cause of the reduced fertility, we examined developing ovaries in *vih^ΔN^* homozygous mutants. The *Drosophila* oocyte undergoes 14 developmental stages (S1–14; figure 2*a*) [8,9]. We found that a large proportion of *vih^ΔN^* egg chambers exhibit severe structural abnormalities as early as in S3 (figure 2*b,c*). While follicular epithelia, with a somatic origin, maintain the intact monolayer, the germline cell membranes that intervene the oocyte and nurse cells (‘oocyte–nurse cell membranes', hereinafter) start collapsing after S3 and subsequently form large aggregates in the centre of the egg chambers (figure 2*b*, white arrows; electronic supplementary material, movies S1 and S2). This phenotype was observed in egg chambers in both homozygous and heterozygous, *vih^ΔN-9^* and *vih^ΔN-4^* mutants (figure 2*b*) and increased with age (electronic supplementary material, figure S2). As this phenotype resembles that of mutations that affect membrane integrity (for example*, rab6^D23D^* mutations) [42], hereinafter, we refer to this phenotype as the ‘membrane phenotype’. Considering its high frequency and severity, we conclude that the membrane phenotype is the main cause of the reduced fertility rate in *vih^ΔN^* mutant females. Because of the higher penetrance of the phenotype in *vih^ΔN-9^* mutants than in *vih^ΔN-4^* mutants, we focus our subsequent analysis on *vih^ΔN-9^* mutants unless stated otherwise.

### 2.2. Centrosome transport is compromised in *vih^Δ^^N^* egg chambers

To determine the primary cause of the membrane phenotype, we examined the *vih^ΔN^* mutant germline earlier than S3 when the disruption of the oocyte–nurse cell membranes becomes apparent. At the start of oogenesis, germline stem cells located at the anterior tip of the germarium divide asymmetrically to generate cystoblasts, which in turn undergo exactly four rounds of mitotic division without complete cytokinesis to form 16 syncytial cytocytes (figure 2*a*). In all the *vih^ΔN-4^* and *vih^ΔN-9^* germline cysts examined, 16 cells were detectable, as in control (figure 2*b*), confirming our result that *vih*^Δ*N*^ mutations do not block mitotic division (electronic supplementary material, figure S1). We next examined the centrosomes within the cyst in *vih^ΔN^* mutants. After the cyst formation, centrosomes within the 16 cells of the cyst migrate intercellularly through ring canals and accumulate in one cell, the presumptive oocyte (figure 2*a*) [7]. We visualized centrosomes by immunostaining against the centriole component Asterless (Asl) [43], and found that, in control S1–3 egg chambers, the majority of centrosomes were located in the presumptive oocyte, in which they subsequently clustered at the posterior region (figure 2*d,e*; electronic supplementary material, movie S3). However, in a large proportion of *vih^ΔN-9^* S1–3 egg chambers, the majority of centrosomes were retained by nurse cells, often adjacent to ring canals (figure 2*d,e*). Even the centrosomes that had reached the oocyte were frequently mispositioned, lingering around in the anterior region (figure 2*d*; electronic supplementary material, movie S4). Thus, germline centrosomes fail to migrate properly from nurse cells to the oocyte in *vih^ΔN^* mutant egg chambers. Importantly, the incidence of this centrosome migration defect in early-stage egg chambers tightly correlates with the appearance of the membrane phenotype in later stages in the same ovarioles: in the *vih^ΔN-9^* ovarioles that exhibited the membrane phenotype after S3, average 9.2 centrosome clusters were present in the S1–3 egg chambers while the ovarioles with no membrane phenotype showed average 3.5 centrosome puncta, comparable to the control (figure 2*e*). This coexistence of the two phenotypes within the same ovarioles suggests a potential causal link between the centrosome transport defect in early egg chambers and the membrane phenotype in later-stage egg chambers in *vih^ΔN^* mutants.

### 2.3. Destabilization of centrosomes interferes with their migration in *vih^Δ^^N^* egg chambers

The mechanism underlying the centrosome transport is largely unknown. Thus, to gain insight into the molecular mechanism, we wished to determine how *vih^ΔN^* mutations impede the centrosome transport. We previously showed that the centrosomes cannot migrate into the oocyte in germline clones with a mutation in *Dynein heavy chain 64C* (*Dhc64c*), suggesting that the function of the minus end-directed microtubule motor dynein is required for the centrosome migration [24]. However, in the *Dhc64c* mutant germarium, the fusome becomes prematurely fragmented during the time when centrosomes migrate [24]. It was observed that centrosomes migrate along the fusome [17,21]. Thus, to test if the centrosome migration defect in the *vih*^Δ*N*^ mutant may be caused by the disruption of the fusome, we examined the fusome structure. Unlike the *Dhc64C* mutations, the morphology of the fusome appears normal in *vih^ΔN-9^* germaria: its branching network that extended throughout the cyst was clearly observed in Region 2 (electronic supplementary material, figure S3A,B), and centrosomes were found attached to the fusome, similar to control (*n* = 17; electronic supplementary material, figure S3A). Thus, it is unlikely that the centrosome migration defect observed in the *vih*^Δ*N*^ mutant germlines is caused by the disruption of the fusome.

**Figure 3.**
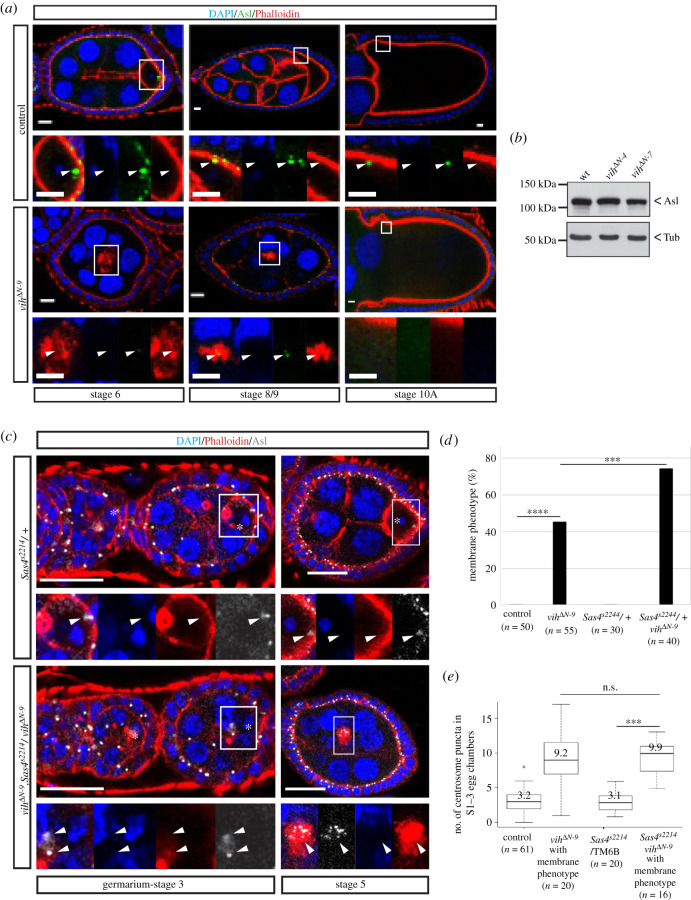
Centrosome stability is decreased in *vih*^Δ*N-9*^ egg chambers. (*a*) S6–10A control and *vih*^Δ*N-9*^ egg chambers with the membrane phenotype, stained for DNA (DAPI) in blue, centrioles (Asl) in green and F-actin (Phalloidin) in red (arrowheads indicate centrioles). (*b*) Western blot of control, *vih*^Δ*N-4*^ and *vih*^Δ*N-9*^ ovary extracts stained with Asl and Tub antibodies. All scale bars equal to 10 µm. (*c*) Germarium-S5 *Sas4^s2214^*/TM6B and *vih*^Δ*N-9*^*, Sas4^s2214^/vih*^Δ*N-9*^ egg chambers stained for DNA (DAPI) in blue, F-actin (Phalloidin) in red and centrioles (Asl) in white (arrowheads indicate centrioles and asterisks the oocyte). (*d*) The quantification of ovarioles that exhibited the membrane phenotype. (*e*) The quantification of centrosome puncta in S1–3 egg chambers, significance determined using a Wilcoxon signed-rank test (*p* < 0.001). All scale bars equal to 10 µm.

We next turned our attention to the germline centrosomes themselves. We noted that the intensity of Asl signals is generally lower in *vih*^Δ*N*^ egg chambers than in control. In addition, we found that Asl signals, hence centrosomes, disappear earlier in *vih*^Δ*N*^ egg chambers than in control: in control egg chambers, a cluster of centrosomes were detectable in the oocyte until S12 before being gradually degenerated, as reported previously [23], whereas, in *vih*^Δ*N-9*^ egg chambers with the membrane phenotype, centrosome signals were reduced after S6 and virtually undetectable after S9 (figure 3*a*). However, the expression levels of Asl proteins in the lysates from *vih*^Δ*N*^ and control ovaries were comparable (figure 3*b*). These data suggest that centrosomes may be structurally unstable in *vih^ΔN^* egg chambers.

We speculate that this destabilization of germline centrosomes observed may underlie the centrosome migration defect in *vih^ΔN^* egg chambers. We reasoned that, if the instability of centrosomes is indeed a cause of the centrosome migration defect, further destabilizing the centrosomes may enhance the occurrence of the centrosome migration failure in the *vih^ΔN-9^* mutants. To test this, we decided to reduce the dosage of Sas4 protein, a core centriole component that is also required for the PCM recruitment during mitosis [44,45], to destabilize germline centrioles. We found that removing one functional copy of *sas4* gene by using a loss-of-function allele (*sas4^s2214^*) significantly increased the proportion of ovarioles exhibiting the membrane phenotype (45–73%, *p* = 0.0002; figure 3*c,d*), concurrently with centrosome migration defects (figure 3*e*). Meanwhile, in the wild-type background, the heterozygous *sas4^s2214^* mutation caused neither of these phenotypes (figure 3*c–e*). This result supports our hypothesis that centrosome instability may underlie the failure in the centrosome transport in the *vih^ΔN^* mutant germline. Furthermore, the persisting coexistence of the centrosome migration defect and the membrane phenotype observed in *sas4^s2214^/+; vih*^Δ*N-9*^ ovaries further supports the possible causality between these two phenotypes.

### 2.4. A reduction of Polo kinase impedes the germline centrosome transport

**Figure 4.**
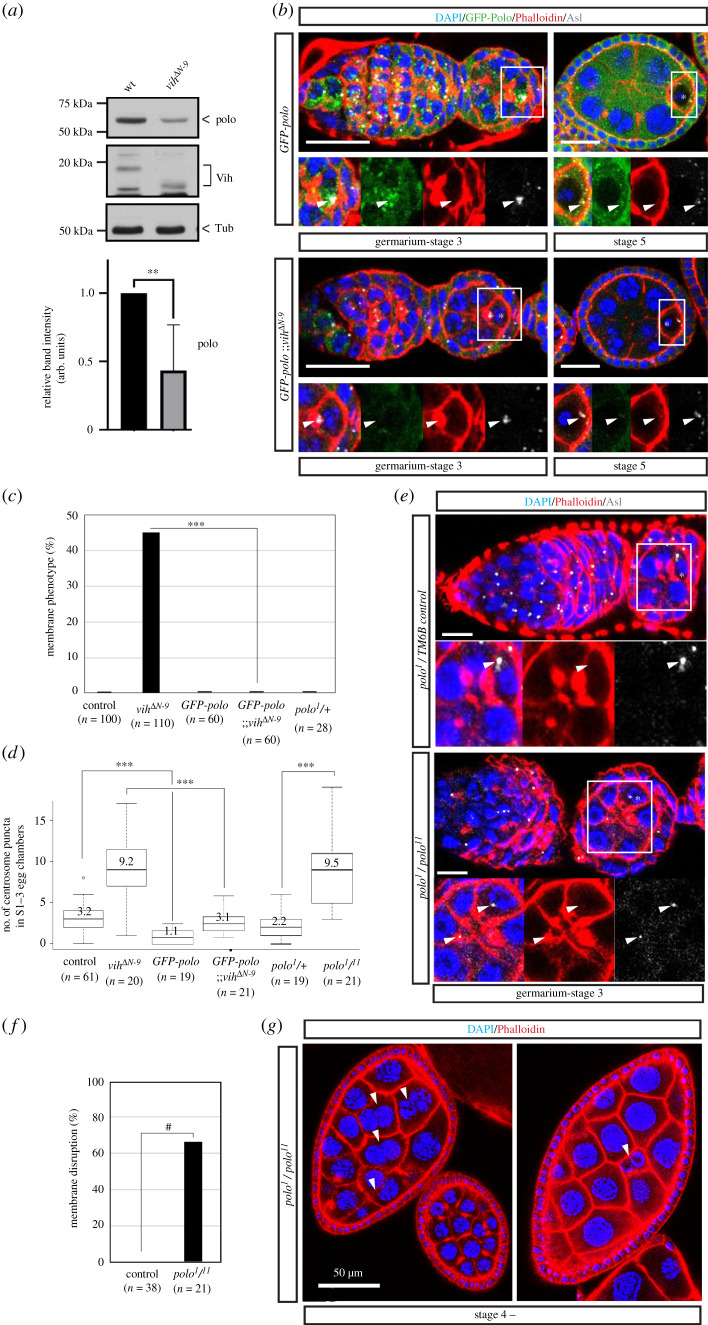
Polo kinase regulates centrosome transport and stability in developing *Drosophila* ovaries. (*a*) Western blot of control and *vih*^Δ*N-9*^ ovary extracts 14 days after eclosion, stained with Vih, Polo and α-tubulin antibodies. The quantifications of the band intensity of Polo protein, indicating the mean normalized values of Polo signals from three independent experiments. The error bar indicates the s.d. A statistical significance of Polo reduction determined using a Student *t*-test (*p* < 0.01) and indicated as asterisks. (*b*) GFP-Polo in control and *vih*^Δ*N-9*^ egg chambers stained for DNA (DAPI) in blue, GFP-Polo in green, F-actin (Phalloidin) in red and centrioles (Asl) in white (arrowheads indicate centrioles and asterisks oocytes). (*c*) The quantification of ovarioles that exhibited the membrane phenotype in indicated genotypes (*n* indicates total sample number in two biological replicates). Significance determined using a Student *t*-test (*p* < 0.001). (*d*) The quantification of ectopic centrosome puncta stained with Asl in S1–3 control, *vih*^Δ*N-9*^, *GFP-polo* and *GFP-polo*;; *vih*^Δ*N-9*^, *polo^1^* / TM6B, and hypomorph *polo* mutant (*polo^1^*/*polo^11^*) egg chambers. Significance determined using a Wilcoxon signed-rank test (*p* < 0.005). (*e*) *polo^1^*/TM6B and *polo^1^*/*polo^11^* egg chambers stained for DNA (DAPI) in blue, F-actin (Phalloidin) in red and centrioles (Asl) in white (arrowheads indicate centrioles and asterisks oocytes). Scale bars equal to 10 µm. (*f*) The quantification of the membrane disruption phenotype. The percentages of control (*w^1118^*, *n* = 38) and *polo^1^*/*polo^11^* (*n* = 39) ovarioles containing mid–late stage (after S4) egg chambers exhibiting disruption of nurse cell membranes were quantified. ^#^*p*-value ≤ 0.0001. (*g*) Examples of nurse cell membrane disruption observed in mid–late stage (after S4) *polo^1^*/*polo^11^* egg chambers stained for DNA (DAPI) in blue, F-actin (Phalloidin) in red. White arrowheads indicate nurse cells with disrupted membranes.

An evolutionarily conserved kinase Polo (Plk1 in human) is a key regulator of centrosomes which recruits the PCM to the centrioles by directly phosphorylating Sas4 [46]. A recent study identified Polo as a key regulator of the stability of centrosomes in the *Drosophila* female germline: it was shown that downregulation of Polo by RNA interference causes premature shedding of the PCM, leading to an early elimination of centriole from the oocyte, while tethering Polo to centrioles stabilizes the centrosomes, allowing their persistence beyond oogenesis, even in the mature egg [23]. Therefore, we suspected that Polo function may be altered in *vih^ΔN^* egg chambers, which causes destabilization of centrosomes. We examined the expression levels of endogenous Polo proteins in *vih^ΔN^* mutant ovaries to find a significant reduction of Polo levels in *vih^ΔN-9^* ovary extracts, compared to control (figure 4*a*; electronic supplementary material, figure S4).

Next, to determine if the reduced Polo levels can account for the *vih^ΔN-9^* phenotypes, we tested whether increasing the expression levels of Polo, by introducing a functional *polo* transgene (*GFP-polo*) [46], could rescue the centrosome migration defect and the membrane phenotype. We found that two copies of *GFP-polo* in the *vih^ΔN-9^* background were able to completely rescue the membrane phenotype (0%, *n* = 60; figure 4*b,c*) and bring the centrosome transport efficiency back to the control level (average 3.1 puncta, *n* = 21; figure 4*b,d*). These data suggest that the reduction of Polo may be the main cause of the centrosome transport defect in the *vih^ΔN^* egg chambers, probably through destabilization of germline centrosomes. It is noteworthy that the wild-type flies carrying *GFP-polo* transgene showed less centrosome puncta (1.1 puncta, *n* = 21; figure 4*d*) than those without the transgene (3.2 puncta, *n* = 61; figure 4*d*) in early egg chambers, suggesting that higher expression of Polo may accelerate the centrosome migration into the oocyte.

This result also suggests that Polo may regulate the centrosome transport during normal oocyte development. To directly test this notion, we investigated the centrosome transport in egg chambers in *polo* hypomorph mutants. The transheterozygous mutants carrying hypomorphic (*polo^1^*) and amorphic (*polo^11^*) alleles are viable but are sterile [47]. *polo^1^/polo^11^* mutant egg chambers exhibited highly pleotropic phenotypes, including mitotic defects of cystoblasts (figure 4*e*). Thus, to specifically address the Polo function in the centrosome migration and later oocyte development, we focused our subsequent analysis on egg chambers containing 16 cells, which indicate the successful completion of four rounds of mitotic division (43%, *n* = 104). In this population, we observed an average of 9.5 centrosome puncta within S1–3 egg chambers (*n* = 21; figure 4*d,e*), indicating a defect in centrosome migration similar to *vih^ΔN^* mutants. Furthermore, we also found that membranes between nurse cell were disrupted in a large fraction of mid-to-late stage egg chambers (66.7%, *n* = 26), although less severe compared to *vih^ΔN^* mutants (figure 4*f,g*). All together, these results strongly suggest that Polo is the key regulator of centrosome migration within the germline cyst, whose protein levels are reduced in the *vih^ΔN^* mutant female germline. Proper transport of centrosomes to the oocyte or normal levels of Polo may be critical to maintain the integrity of nurse cell membranes during the later stage of oocyte development.

### 2.5. *Vih^Δn^* mutations cause the upregulation of APC/C, leading to premature Polo degradation

**Figure 5.**
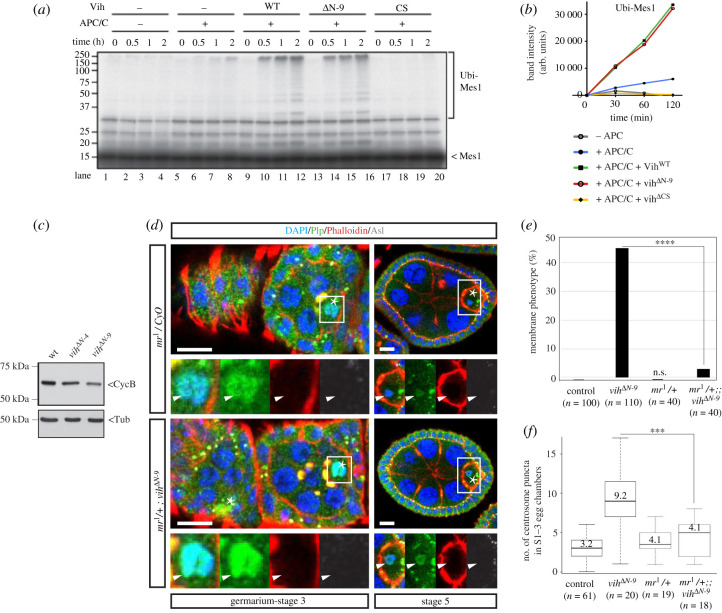
APC/C^Vih^ prematurely targets Polo in *vih*^Δ*N*^ mutant ovaries. (*a*) Reconstituted *in vitro* ubiquitination assays using purified *Drosophila* APC/C and ^35^S-labelled *S. pombe* Mes1 as a substrate. No Vih protein was added in lanes 1–8 while recombinant Vih^WT^, Vih^ΔN-9^ and dominant-negative Vih^ΔCS^ protein was supplemented in lanes 9–12, 13–16 and 17–20, respectively. (*b*) The quantification of ubiquitinated Mes1 signals in *c*. (*c*) Western blot of control, *vih*^Δ*N-4*^ and *vih*^Δ*N-9*^ ovary extracts, blotted with CycB and α-tubulin antibodies. (*d*) Representations of Germarium-S5 *mr^1^*/CyO and *mr^1^/+; vih*^Δ*N-9*^ egg chambers stained for DNA (DAPI) in blue, PCM (Plp) in green, F-actin (Phalloidin) in red and centrioles (Asl) in white (arrowheads indicate Plp signals on oocyte nuclei and asterisks oocytes). (*e*) The quantification of ovarioles that exhibited the membrane phenotype in indicated genotypes (*n* indicates the total sample number in two biological replicates). (*f*) The quantification of ectopic centrosome puncta co-stained with Asl in S1–3 control, *vih*^Δ*N-9*^, *mr^1^*/CyO and *mr^1^/+; vih*^Δ*N-9*^ egg chambers, significance determined using the Student *t*-test (*p* < 0.001).

We next asked how *vih^ΔN^* mutations cause the Polo reduction. As Polo orthologues are known to be targets of the APC/C-dependent degradation in other species including human cells [48,49], we speculated that Polo might be prematurely degraded by APC/C in *vih^ΔN^* mutants. It was previously shown that the N-terminal extension conserved among the Ube2C family of E2 enzymes is not essential for the E2 catalytic activity of human Ube2C (also known as UbcH10) but rather important for negatively regulation of the APC/C by human Ube2C [37,41]. We therefore hypothesized that the *vih*^Δ*N*^ mutations may specifically abolish the APC/C-inhibitory function of Vih, thereby causing APC/C upregulation *in vivo*. We confirmed that Vih*^Δ^*^N^ is indeed as catalytically active as Vih^WT^ in a reconstituted APC/C-dependent ubiquitination assay using *Drosophila* APC/C that was purified from *Drosophila* embryos (see Methods for the detailed protocol; figure 5*a,b*): the ubiquitination activity of APC/C-Vih^Δ^^N-9^ (lanes 13–16, figure 5*a*) on a model APC/C substrate, Mes1, was comparable to APC/C-Vih^WT^ (lanes 9–12) while catalytically inactive Vih*^Δ^*^CS^ (lanes 17–20) did not support ubiquitination of Mes1.

Next, we monitored APC/C activity *in vivo* by analysing the endogenous levels of CycB, a known APC/C substrate, in ovaries. We observed an apparent reduction in CycB levels in *vih^ΔN^* ovary extracts compared to control, in support of the upregulation of APC/C activity in *vih^ΔN^* ovaries (figure 5*c*). Finally, to test if the *vih^ΔN^* phenotypes are caused by deregulated APC/C activity, we reduced APC/C activity by halving the dose of the gene encoding the APC/C catalytic subunit, *morula* (*mr*), in the *vih^ΔN-9^* homozygous background. We found that the heterozygous *mr* mutation strongly suppressed both the membrane phenotype and the centrosome migration defect (figure 5*d–f*). Similar suppression of the *vih^ΔN-9^* phenotypes was also observed by over-expression of the canonical APC/C substrate Cyclin B, which inhibits the degradation of other APC/C substrates through competition for the binding sites on the APC/C (data not shown). Collectively, these data strongly suggest that *vih^ΔN^* mutations cause the upregulation of APC/C in the germline cyst, probably through the loss of negative regulation of the APC/C by Vih. This deregulation of APC/C leads to the destabilization of its substrate Polo, which in turn impedes the centrosome transport.

### 2.6. The centrosome migration may be important for the maintenance of oocyte fate

**Figure 6.**
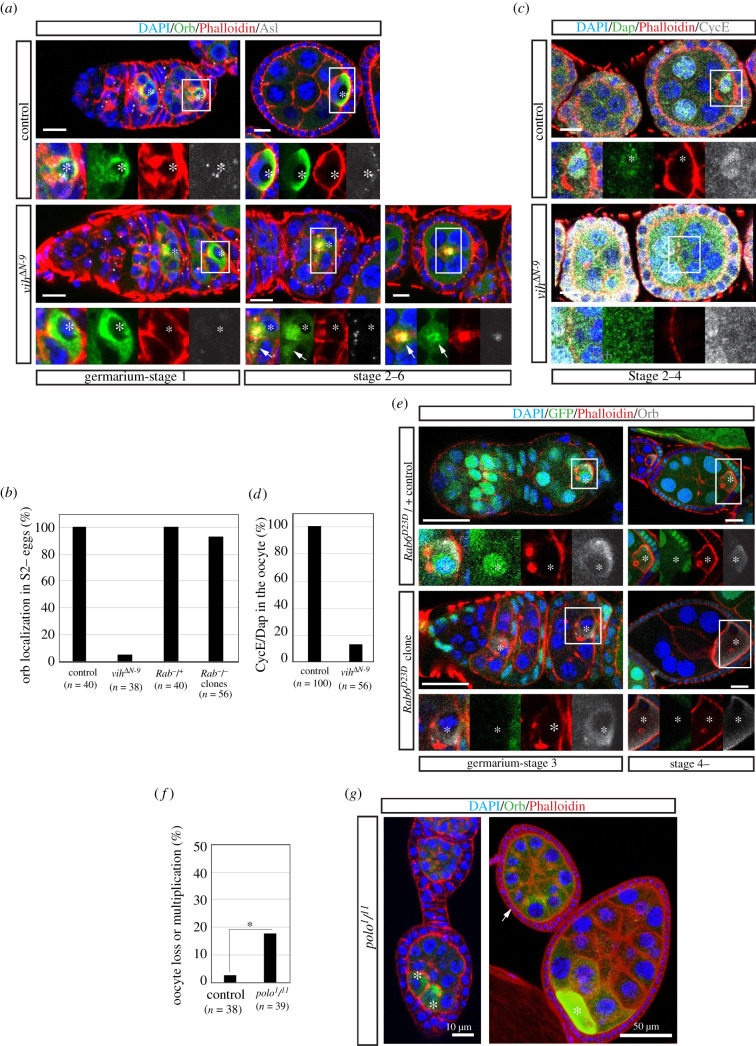
Proper control of Vih activity is necessary for the maintenance of oocyte fate. (*a*) Germaria-S5 control and *vih*^Δ*N-9*^ egg chambers stained for DNA (DAPI) in blue, Orb in green, F-actin (Phalloidin) in red and centrioles (Asl) in white (arrows indicate mislocalized Orb signals and asterisks oocytes). (*b*) The quantification of ovarioles or egg chambers that exhibited Orb mislocalization after S2 in indicated genotypes (*n* indicates the total sample number in two biological replicates). (*c*) S2–S4 control and *vih*^Δ*N-9*^ egg chambers stained for DNA (DAPI) in blue, Dap in green, F-actin (Phalloidin) in red and CycE in white (asterisks indicate oocytes). (*d*) The quantification of ovarioles that exhibited CycE/Dap accumulation in the oocyte during Stage 2–4 (*n* indicates the total sample number in two biological replicates). (*e*) Representations of egg chambers containing *Rab6^D23D^*/CyO or *Rab6^D23D^* germline clones (marked by the presence of the lack of GFP signals, respectively), stained for DNA (DAPI) in blue, GFP in green, F-actin (Phalloidin) in red and Orb in white (asterisks indicate the oocyte). All scale bars equal to 10 µm. (*f*) The quantification of control and *polo^1^*/*polo^11^* ovarioles that contain egg chambers with no or more than one oocytes. Asterisks indicate the *p*-value: *≤ 0.05. (*g*) Examples of egg chambers with no or more than one oocytes in *polo^1^*/*polo^11^* ovarioles. White asterisks indicate oocytes. White arrow indicates the egg chambers without detectable oocytes with Orb dispersed within the egg. The scale bars indicate 10 µm (left) and 50 µm (right).

As shown above, *vih*^Δ*N*^ mutations prevent the centrosome transport without affecting the fusome structure, unlike previously reported *Dhc64c* mutations [50] (figure 2*d,e*; electronic supplementary material, figure S3). Thus, further characterization of *vih*^Δ*N*^ mutant phenotypes may provide a novel insight into the specific role of the centrosome migration in the *Drosophila* oocyte development. We examined mid-to-late stages (S3 onwards) of *vih^ΔN^* egg chambers and noted that many *vih^ΔN^* egg chambers have no distinguishable ‘oocytes’ (based on the size and the morphology of the nucleus). We therefore tested if an oocyte is properly selected among the 16 cells of the cyst in *vih^ΔN^* egg chambers by staining Orb, an oocyte fate marker that is localized in the oocyte cytoplasm [12]. In all the *vih^ΔN-9^* S1 egg chambers examined, including those in the ovarioles displaying the membrane phenotype, Orb accumulated strongly in the cytoplasm of a single cell in each cyst, indicating that the oocyte is properly specified within a cyst in *vih^ΔN^* egg chambers (figure 6*a*). However, in 95% of *vih^ΔN-9^* ovarioles exhibiting the membrane phenotype (*n* = 38), Orb failed to remain localized in the oocyte after S3 and spread weakly through the entire cytoplasm, frequently forming ectopic foci around centrosome/F-actin aggregates in the middle of the egg chamber (figure 6*a,b*). We also checked whether oocytes keep arrested in meiotic prophase I in *vih^ΔN-9^* egg chambers, by analysing the expression of the cell cycle markers, Dacapo (Dap) and Cyclin E (CycE) [51]. In all control egg chambers examined, both cell cycle markers localized to the germinal vesicle in the oocyte from early to late oogenesis (*n* = 100; figure 6*c,d*). By contrast, in 86% of *vih^ΔN-9^* egg chambers (*n* = 56), there was no obvious Dap and CycE accumulation within the germinal vesicle after S3 and the oocyte appeared to become polyploid through endocycle as nurse cells (judged by its nuclear size) (figure 6*c,d*). Thus, the oocyte is initially specified but subsequently lose its identity in the *vih^ΔN-9^* germline.

Since we also observed the centrosome migration defect in *polo* mutant ovaries (*polo^1^/polo^11^*, figure 4*d,e*), we also examined the maintenance of oocyte identity in *polo* mutant egg chambers by analysing Orb localization. We found that a small but statistically significant fraction of egg chambers (after S4) had no oocytes (cells with Orb accumulation) or more than one oocytes in the *polo* ovaries (18%, *n* = 39, *p* = 0.0146; figure 6*f,g*). Together, these results suggest that the migration of centrosomes into the oocyte may be critical for the maintenance of oocyte identify after it is specified within the cyst in the germarium.

As shown above, nurse cell membranes are often disrupted in *vih^ΔN^* and *polo* mutant egg chambers (figures 2*b,c* and 4*f,g*), it may be possible that the loss of oocyte fate in these mutant egg chambers is caused merely by the disruption of the oocyte–nurse cell membranes (i.e. the membrane phenotype), allowing the dispersal of oocyte determinant factors from the oocyte cytoplasm. To test whether membrane breaking is indeed accountable for the oocyte loss, we examined egg chambers carrying a mutation in a small GTPase Rab6 (*rab6^D23D^*), which causes the membrane disruption in the germline cyst, due to defective membrane trafficking [42]. We generated homozygous *rab6^D23D^* mutant germline clones using the FLP/FRT system [52]. As reported previously, oocyte–nurse cell membranes as well as membranes between nurse cells started collapsing in the mutant egg chambers after S3, similar to *vih^ΔN^* egg chambers (figure 6*e*). Nevertheless, in the majority of the *rab6^D23D^* mutant egg chambers including those in which the oocyte–nurse cell membranes were partially raptured, oocytes were still clearly identifiable by their compact nuclear size and Orb accumulation around the nuclei until late stages (93%, *n* = 56, figure 6*b,e*). This is in stark contrast to *vih^ΔN-9^* and *polo^1^/polo^11^* mutants where Orb localization is almost entirely lost in egg chambers (figure 6*a,b,f,g*). Thus, although we cannot completely rule out the possibility, membrane breaking is unlikely to be accountable, at least solely, for the oocyte fate loss in *vih^ΔN^* and *polo* mutant egg chambers.

### 2.7. The centrosome transport may promote the formation of the polarized microtubule network in the germline cyst

The tight correlation between centrosome migration defects, membrane disruption and oocyte fate loss observed in *vih^ΔN^* and *polo* mutant germlines suggests a possible causal relationship between these phenotypes. It is known that, shortly after the oocyte is specified within a cyst, microtubules start to accumulate in the oocyte and form a polarized network that emanates from the oocyte and extend through the whole cyst. This oocyte-centric polarized network is believed to be required for oocyte maturation by mediating the directed transport of various proteins and RNAs, including oocyte fate markers, in developing egg chambers [9,19,20]. It has been postulated that, while the microtubule nucleation activity of germline centrosomes appears to be inactivated during migration, it may be reactivated in the oocyte and promote the formation of the polarized microtubule network [6,17,21]. We therefore hypothesized that the failure in centrosome migration into the oocyte may interfere with the establishment of the oocyte-centric polarized microtubule network within the egg chamber. This can explain the correlation between the centrosome migration defect and the loss of oocyte identity in *vih^ΔN^* and *polo* mutant ovaries.

**Figure 7.**
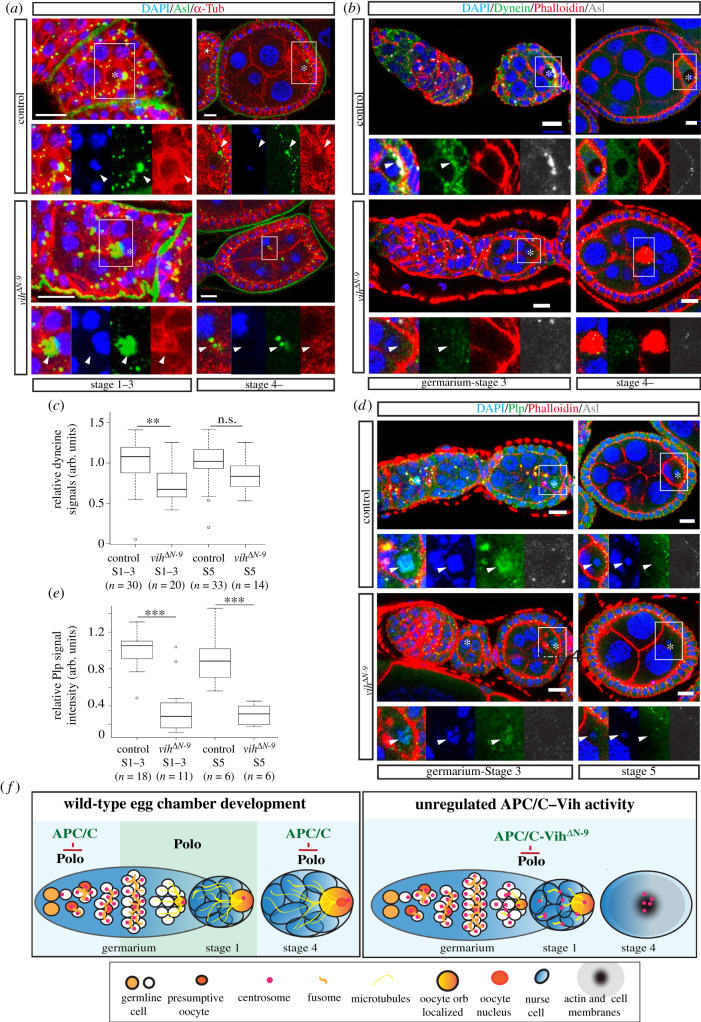
Centrosome transport facilitates the formation of the polarized microtubule network in the egg chamber. (*a*) Control and *vih*^Δ*N-9*^ S1 and S4–6 egg chambers stained for DNA (DAPI) in blue, centrioles (Asterless) in green and microtubules (α-Tubulin) in red (arrowheads indicate centrioles and asterisks oocytes). (*b*) Control and *vih*^Δ*N-9*^ Germarium-S3 and S4–6 egg chambers stained for DNA (DAPI) in blue, Dynein in green, F-actin (Phalloidin) in red and Asl in white (arrowheads indicate dynein signals and asterisks indicate oocytes). (*c*) The quantification of Dynein staining on the oocyte nucleus S1–3 and S4–6 for control and *vih*^Δ*N-9*^ egg chambers with an oocyte nucleus, significance determined using a Welch's unbiased *t*-test (S1–3: *p* = 1.4 × 10^−3^) and a Wilcoxon signed-rank test (S4–6 oocyte: 1 × 10^−2^ < *p* < 5 × 10^−1^). (*d*) Germaria-S3 and S5 control and *vih*^Δ*N-9*^ egg chambers stained for DNA (DAPI) in blue, PCM (Plp) in green, F-actin (Phalloidin) in red and centrioles (Asl) in white (asterisks indicate oocytes). Scale bars equal to 10 µm. (*e*) The quantification of Plp staining on the oocyte nucleus S1–3 and S5 control and *vih*^Δ*N-9*^ egg chambers, significance determined using a Wilcoxon signed-rank test (S1–3: 1 × 10^−3^ < *p* < 5 × 10^−3^; S5: 1 × 10^−2^ < *p* < 5 × 10^−1^). (*f*) Model for APC/C regulation. APC/C activity needs to be turned off after the last round of germline mitosis in order to stabilize Polo levels thereby enabling the centrosomes to remain stable and be transported to the oocyte. When there is greater APC/C activity then Polo is degraded precociously and the egg chamber becomes destabilized.

To test this hypothesis, we first monitored microtubule structures in fixed preparations of *vih^ΔN^* egg chambers. In control S4–6 egg chambers, microtubules were highly accumulated in the oocyte and some pools of the microtubules appeared to be nucleated in the vicinity of centrosome clusters, which were located in the posterior region of the oocyte (figure 7*a*, control). By contrast, in all S4–6 *vih^ΔN-9^* egg chambers exhibiting the membrane phenotype, no specific accumulation of microtubules was observed within egg chambers and only short microtubule bundles grew from some of ectopic centrosomes (figure 7*a*, *vih*^Δ*N-9*^, Stage 4–). However, in all early S1 egg chambers before membranes start collapsing, the accumulation of microtubules in the oocyte was visible in both control and *vih^ΔN^* mutants (figure 7*a*, Stages 1–3), suggesting that microtubules start nucleating in the oocyte and start forming a network in *vih^ΔN^* egg chambers. Thus, we next assessed whether the microtubule network is properly polarized within the cyst by examining the localization of the minus end-directed microtubule motor dynein (Dhc64C) [53]. As reported previously [53], in control egg chambers, Dhc64C signals were accumulated at the periphery of the germinal vesicle during S1–3 and continued being enriched in the oocyte cytoplasm until late stages (figure 7*b,c*), indicative of the polarization of microtubules with their minus end oriented to the oocyte. However, in *vih^ΔN-9^* S1–3 egg chambers (with still detectable oocyte–nurse cell membranes), we found a significant reduction in the Dhc64C localization in the oocyte (figure 7*b,c*), indicating reduced microtubule polarization. Thus, in the *vih^ΔN^* egg chambers, although the microtubule network begins to form, it is not properly (at least not fully) polarized. Since the polarized microtubule network is crucial for the directed intercellular trafficking of various materials, including oocyte determinants, between the oocyte and nurse cells [9,19,20], this reduced microtubule polarization is likely to account for the subsequent loss of oocyte fate in *vih^ΔN^* egg chambers.

### 2.8. Centrosome migration may be required for concentrating and confining microtubule nucleation activity to the oocyte in the egg chamber

The above result also points to the potential role for the centrosome transport in facilitating the establishment of the oocyte-centric polarized microtubule network within the egg chamber. Thus, we next wished to investigate the possible underlying mechanism through which the transport of germline centrosomes promotes the polarization of the microtubule network.

It is known that the microtubule-organizing activity of the centrosome is mediated by the PCM [3,4]. Thus, the PCM may play a role in the formation of the oocyte-centric polarized microtubule network. Therefore, we examined the state of the PCM on germline centrosomes in *vih^ΔN^* egg chambers, by staining the Pericentrin-like protein (Plp), a PCM component of both the interphase and mitotic centrosome that is located adjacent to the centriole wall and helps recruit other PCM proteins [3,54]. Consistent with a previous report [23], we observed that Plp labelled centrosomes until late stages of oogenesis in both control and *vih^ΔN-9^* egg chambers. However, in addition to these centrosomal signals, we unexpectedly observed a strong accumulation of Plp on the oocyte nucleus in the control egg chambers, which appeared before S1 and tapered off between S4 and S6 (figure 7*d*). Although our observation was the first for endogenous Plp proteins, a similar localization pattern on the oocyte nucleus was previously reported for over-expressed GFP-tagged C-terminal domain of Plp (PACT domain) and endogenous γ-tubulin [55–57]. We found that, compared to the control, these Plp signals on the oocyte nucleus were significantly reduced in S1–3 *vih^ΔN-9^* egg chambers, which still retained clearly visible membranes between the oocyte and adjacent nurse cells (figure 7*d,e*). It was previously reported that microtubules also nucleate on the surface of oocyte nuclear membranes and contributes to the posteroanterior movement of the oocyte nucleus in S6–7 egg chambers [55,58]. Thus, the migration of the centrosomes into the oocyte may facilitate a transfer of some pool of PCM to the oocyte nucleus, enabling the oocyte nucleus to act as an MTOC. Such MTOC activity of the oocyte nucleus may contribute to the formation of the robust oocyte-centric polarized microtubule network.

The above observations suggest a potential role of the centrosome transport that promotes the formation of the microtubule network by transferring PCM to the oocyte nucleus. However, this model is somewhat contradictory to the earlier observation made by Stevens *et al*. [22] using homozygous *sas4* mutant flies (note that this study used homozygous *sas4* mutants, whereas the heterozygous *sas4* mutation was used in figure 3, keeping one copy of the wild-type *sas4* gene) that, in the ovaries where the vast majority of germ cells (approx. 80%) do not have detectable centrosomes, the oocyte develops normally. To reconcile this apparent controversy, we hypothesize that the centrosome transport may facilitate the establishment of the oocyte-centric microtubule network through two functionally redundant processes: first, bringing centrosomes into the oocyte, together with the PCM (figure 7*d,e*) and, second, removing centrosomes from nurse cells. Centriole loss would affect the first process but not the other.

Based on this hypothesis, we next examined ectopic centrosomes that fail to move to the oocyte and remain in nurse cells. We found that Plp signals were also observed on the mislocalized centrosomes in nurse cells both in control and *vih^ΔN-9^* mutant egg chambers (electronic supplementary material, figure S5A). Short microtubule bundles nucleate at ectopic centrosomes in *vih^ΔN-9^* egg chambers (figure 7*a*). To assess whether mislocalized centrosomes indeed nucleate microtubules, we performed live imaging of egg chambers expressing GFP-fused microtubule plus-end tracking protein EB1 [59] and examined the formation of EB1 comets at individual centrosomes. To avoid pleiotropic effects, we selected S4–5 *vih^ΔN-9^* egg chambers that did not display the membrane phenotype. We observed microtubule nucleation at mislocalized centrosomes in nurse cells in *vih^ΔN-9^* egg chambers, as well as a rare population of mislocalized centrosomes in control egg chambers (electronic supplementary material, figure S5B and movies S5 and S6). Thus, after being kept inactive during the migration of the centrosomes [21], the activity of the centrosomes appears to be reactivated to start nucleating microtubules ectopically in nurse cells. These ectopic MTOCs, in large numbers, may interfere with the formation of the oocyte-centred microtubule network.

To test a potential adverse effect of microtubule nucleation at ectopic centrosomes, we inhibited microtubule polymerization in *vih^ΔN-9^* mutant egg chambers by feeding flies the microtubule destabilizing drug colcemid. Egg chambers from colcemid-fed flies showed clear patterning defects at later stages both in control and *vih^ΔN-9^* mutant ovaries, however, did not affect centrosome migration, in accordance with previous reports [17,24]. Thus, we could not directly assess the effect of colcemid treatment on the maintenance of oocyte fate (Orb accumulation) in *vih^ΔN^* mutant ovaries. Therefore, we examined its effect on the membrane phenotype. We found that, in colcemid-fed *vih^ΔN-9^* mutants, the percentage of egg chambers with the membrane phenotype was partially reduced compared to untreated flies (from 45 to 30%, *p* = 0.021; electronic supplementary material, figure S5C,D), suggesting the involvement of microtubule nucleation at ectopic centrosome in this phenotype.

Together, these results suggest the critical importance of centrosome migration for oocyte development in two ways: first, to facilitate the formation of oocyte-centric microtubule network and, second, to prevent microtubule nucleation at ectopic nurse cells centrosomes.

## 3. Discussion

The intracellular transport of germline centrosomes following cyst formation is observed in diverse animals from insects to mammals, suggesting its functional significance in oogenesis. However, its exact role, as well as the underlying mechanism directing this event, remains unclear. This is in part due to difficulty specifically manipulating this process without affecting other concurrent events that influence oocyte specification [24,25]. In this present study, by using CRISPR-mediated genome editing in *Drosophila*, we created novel mutants in *vih* gene, in which the centrosome migration is impeded with the intact fusome formation and proper initial oocyte specification. By using these mutants, we identified a molecular pathway controlling the centrosome transport, shedding a new light into the role of the centrosome migration in oocyte development.

Our study identified an evolutionarily conserved kinase Polo/Plk1 as a key regulator of the centrosome migration in the *Drosophila* female germline. Polo/Plk1 has been established as a critical regulator of the centrosome in both mitotic and postmitotic cells. Polo/Plk1 recruits PCM components to the centrosome through phosphorylation of centrosome components, which is essential not only for the microtubule nucleation activity of centrosomes but also critical for the stability of centrioles [23,46]. We showed that the expression levels of Polo were significantly decreased in *vih*^Δ*N*^ mutant ovaries and that increasing the *polo* gene dosage fully rescued the centrosome migration defects and the membrane phenotype in *vih*^Δ*N*^ mutant germlines (figure 4*b–d*). Moreover, *polo* mutant females also exhibited a defect in the centrosome migration as well as the disruption of nurse cell membranes, similar to *vih*^Δ*N*^ mutants (figure 4*c–g*). Together, these results strongly suggest the critical importance of Polo in the regulation of the intercellular migration of centrosomes in the *Drosophila* female germline (figure 7*f*). However, our study could not clarify the downstream molecular events through which Polo directs the centrosome migration, including its direct target required for the migration. Our data suggest that centrioles become destabilized in *vih*^Δ*N*^ mutant germlines (figure 3*a,b*) and it was recently shown that the local level or activity of Polo at centrioles is a rate-limiting factor for centriole degeneration in the germline cyst/egg [23]. Thus, one plausible model for the role of Polo is that Polo activity may be required to retain PCM on migrating germline centrioles that ensures the structural integrity and physical strength of the centrioles during migration. To support this model, further destabilization of centrioles, by the reduction of a core centriole component Sas4, increased the incidence of centrosome migration failure of *vih*^Δ*N-9*^ mutants (figure 3*c–e*). Alternatively, as centrosomes migrate along the fusome [17,21], some PCM component recruited by Polo may be directly involved in the interaction between germline centrioles with the fusome. The fusome is decorated with stable acetylated microtubules through the function of the spectraplakin Shot, which is also essential for centrosome migration [25]. The PCM component may directly bind the stable microtubules on the fusome. It is also possible that Polo may regulate centrosome migration independently of the PCM. It was previously shown that, in *Drosophila* ovaries, Polo binds BicD, a cargo adaptor protein that interacts with the microtubule motor dynein [60]. Further investigation is clearly needed to define the role of Polo in the centrosome transport.

Our study also highlights APC/C, the major ubiquitin ligase in cell cycle control, as an upstream regulator of Polo in its function in centrosome migration (figure 7*f*). Polo/Plk1 has been shown to be an APC/C target in diverse organisms from yeast to humans [48,61]. Our *in vivo* data and the data from previous *in vitro* studies on human Vih orthlogue, Ube2C [37,41], suggest that *vih*^Δ*N*^ mutations may disrupt negative regulation of APC/C by Vih through the function of its conserved N-terminal extension, which causes precocious or excess Polo degradation in the germline cyst (figure 7*f*). The rapid and local control of Polo activity is possibly only achievable by proteolytic degradation; transcriptional regulation of *polo* mRNA levels would not allow for a rapid alteration of local protein levels. The APC/C-dependent proteolysis may be critical to fine-tune Polo levels in order to couple the centrosome behaviour to cell cycle regulation in the *Drosophila* female germline. After the completion of mitotic division of cystoblasts, APC/C activity must be turned off to allow Polo accumulation for the centrosome migration and to maintain a pre-meiotic arrest of the oocyte [62]; it then turns back on to degrade Polo to eliminate centrioles from the oocyte and also to maintain endocycling of nurse cells [23,63] (figure 7*f*). Given the conserved roles of the APC/C and Polo kinase, our findings are likely to translate to many other organisms.

Our detailed characterization of the phenotypes in *vih*^Δ*N*^ mutant egg chambers suggests a critical role of the centrosome transport in *Drosophila* oogenesis. Previous studies showed that the activities of dynein and Shot are required for centrosome migration. However, in addition to centrosome migration, dynein mutations also affect the fusome structure and *Shot* mutations prevent the accumulation of microtubules and Orb in a presumptive oocyte, obscuring the specific role of centrosome migration in oocyte specification [24,25]. In *vih*^Δ*N*^ mutant egg chambers, which we created by CRISPR technique, the fusome structure appears normal and Orb, microtubules and specific cell cycle proteins (Dap and CycE) all initially accumulate in the oocyte (figures 6 and 7; electronic supplementary material, figure S3). Thus, in agreement with Stevens *et al.* [22], these results suggest that the transport of centrosomes is dispensable for oocyte specification. However, to our surprise, we found that the majority of *vih*^Δ*N*^ mutant egg chambers eventually lose the oocyte: the oocyte does not retain Orb and becomes polyploid, like nurse cells (figure 6). We also observed the loss or multiplication of the oocyte in *polo^1/11^* mutant egg chambers, pointing to a critical role of the centrosome migration in the maintenance of oocyte identity. Although this result may appear contradictory with the results in the aforementioned study [22], it is not. In *vih*^Δ*N*^ mutant egg chambers, centrioles are absent (more precisely, reduced) in the oocyte, but, unlike homozygous *sas4* mutants, used by the previous study [22], centrioles are abnormally present in nurse cells. Thus, the phenotypes in *vih*^Δ*N*^ mutants are the consequences of germline centrosomes being misplaced, not eliminated entirely. Like our observations in *vih*^Δ*N*^ mutants, there are indeed many examples in which ectopic centrosomes, not the loss of centrosomes, cause serious consequences, including tumorigenesis, as reported in mice over-expressing Ube2C [38]. Therefore, our results extend beyond the specific role of centrosomes in *Drosophila* oocyte development to give insight into the roles and underlying mechanism of centrosome regulation across tissues and organisms.

How does centrosome migration control oocyte maintenance? Although our present study cannot provide a definitive answer to this question, our data suggest that the migration of centrosomes into the oocyte may be crucial for the formation of the oocyte-centric polarized microtubule network within the egg chamber, which is considered vital for the maintenance and maturation of the oocyte, mediating directed intercellular trafficking of proteins, RNA and organelles between/within the oocyte and nurse cells. In *vih*^Δ*N*^ mutant egg chambers, even those that still retain visible nurse cell–oocyte membranes, we observed reduced accumulation of the minus end-directed microtubule motor dynein in the oocyte, which indicates the weakening of microtubule polarization within the cyst. Interestingly, we observed that an interphase PCM component Plp temporally accumulates on the oocyte nucleus in early-stage egg chambers, which is significantly reduced by vih^*ΔN*^ mutations (figure 7*d,e*). It was previously shown that the oocyte contains an MTOC, which nucleates a polarized microtubule network during early stages of oocyte development [17,21]. It was also shown that the oocyte nucleus itself acts as an MTOC after S6 [55,58]. Thus, once centrosomes have reached the oocyte, a fraction of PCM, including Plp, may be transferred from the centrosomes over to the oocyte to confer the oocyte nucleus the microtubule nucleation activity. In addition, we also showed that the centrosomes that fail to reach the oocyte start nucleate microtubules ectopically in nurse cells (figure 7; electronic supplementary material, figure S4B and movies S5 and S6), which if present in a large number, is likely to interfere with proper microtubule organization within the cyst. To support this, we showed that the inhibition of microtubule polymerization by colcemid partially suppresses the membrane phenotype in *vih^ΔN^* mutants (electronic supplementary material, figure S5C,D). Thus, centrosome migration may also be important to prevent ectopic microtubule nucleation in nurse cells. Based on these observations, we hypothesize that the intracellular transport of the centrosomes may facilitate the formation of the oocyte-centred microtubule network through the two redundant mechanisms: by efficiently transferring microtubule nucleation activity to the oocyte, and by preventing microtubule nucleation in nurse cells. However, further studies are clearly needed to establish this model and to elucidate the molecular mechanisms underlying the connection between centrosome migration and the formation of the polarized microtubule network in the *Drosophila* female germline.

## 4. Material and methods

### 4.1. Drosophila stocks

All stocks were maintained at 25°C. All CRISPR/Cas9 engineered stocks were created as detailed below. The nos-cas9 and act-cas9 stocks were a kind gift from Fillip Port. The transgenic gRNA flies were created using either *y^1^ sc^1^ v^1^ P{nos-phiC31\int.NLS}X; P{CaryP}attP2* (BDSC 25710) or *y^1^ v^1^ P{nos-phiC31\int.NLS}X; P{CaryP}attP40* (BDSC 25709). The controls used were either *w^1118^* or *w^+O^*, except for the internal control which was w*; P{w^+mC^ *=* *Ubi-GFP.D}61EF P{w^+mW.hs^* *=* *FRT(w^hs^)}2A* (BDSC 1626). The FRT chromosome that the *vih*^Δ*N-4*^ allele was created on was w*; P{w^+mW.hs^ *=* *FRT(w^hs^)}2A* (BDSC 1997). The FRT chromosome that was used for the *Rab6^D23D^* germline clones was w*; P{w^+mC^ *=* *Ubi-GFP(S65 T)w^hs^)nls} 2LP{ry^+t7.2^* *=* *neoFRT}40A/CyO* (BDSC 5629). The *vih* deficiencies used for the complementation test were *w^1118^; Df(3 L)BSC380/TM6C, Sb^1^ cu^1^ (BDSC-24404)* and *w^1118^; Df(3 L)ED4483, P{3’.RS5* *+* *3.3'}ED4483/TM6C, cu^1^* Sb^1^ (BDSC-8070). The ubiquitously expressed transgenic fly lines we generated for this study: *P{Ubi-vih-WT}*, *P{Ubi-vih^ΔN-9^}* and *P{Ubi-vih^ΔCS^}*. The *mr* alleles were obtained from the Bloomington Drosophila Stock Center (BDSC), *e(mr)^1^;mr^2^/SM6a* (BDSC-4535) and *px^1^ bw^1^ mr^1^ sp^1^/In(2LR)bw^V1^, ds^33 k^ bw^V1^* (BDSC-380). The GFP-polo stock expressing GFP-fused Polo under the endogenous promotor was obtained from Claudio Sunkel [64]. The *w; rab6^D23D^, FRT40A/Cyo* was a kind gift from Anna Ephrussi. The *Ubi-cycB-GFP* was a kind gift from Jordan Raff. The *polo^1^* and *polo^11^* alleles were a kind gift from Adelaide Carpenter. The *Sas4^s2214^* allele was a kind gift from Paul Conduit. The *asl:YFP* allele was a kind gift from C. González. The *Eb1:GFP* allele originated from the Uemura laboratory.

### 4.2. Generation of the transgenic vih mutants

The gRNA sequenced used was CGAGCAAAGTGGTGGAGCAG. The target site was selected in order to direct Cas9 cleavage to just after the 5′ start methionine codon of the transcript. The CRISPR Optimal Target Finder (http://tools.flycrispr.molbio.wisc.edu/targetFinder/) was used to design the 20mer target sequence. Off-target assessment was done with this program and the target site was chosen because it did not have any homologous sites anywhere else in the genome. The gRNA plasmid pCFD3 (a gift from Fillip Port) was used to create the gRNA transgenic flies [40]. The oligos were cloned directly into the pCFD3 vector for the production of the transgenic gRNA line. All fly embryos were injected by the Cambridge Genetic Fly Facility, using standard procedures. The gRNA expression plasmid was inserted into the genome using the Phi31C system at the attP40 site.

For each experiment, transgenic *cas9* virgin females were crossed to U6-gRNA-expressing males carrying the gRNA plasmid insertion, similar to previous studies [40]. Individual females resulting from this cross were subsequently crossed to a balancer stock to remove the Cas9 expression and the resulting single females were again crossed to the balancer stock to generate a stable fly line. Therefore, the other chromosomes that were present during the mutagenesis were eliminated during stock creation. When the alleles were created in an FRT background, the gRNA line was first balanced and crossed to flies containing the FRT chromosome; males of this line were then crossed to transgenic *cas9* virgin females and carried through the normal procedure.

### 4.3. CRISPR allele characterization and additional phenotypic data

In order to identify CRISPR/Cas9-induced mutations, genomic DNA was isolated from flies by crushing them in 200 µl of BufferA (100 mM Tris pH 9.0, 100 mM EDTA, 1% SDS), and incubating the lysate at 65–70°C for 30 min. The lysate was chilled on ice for 5 min, before adding 30 µl of 7.5 M NH_4_-acetate. The lysate was then mixed gently, chilled on ice for 20 min and centrifuged for 10 min at 13 000 rpm. The supernatant was transferred to a new tube and spun again for 10 min. The supernatant was again transferred to a new tube, mixed gently with 150 µl of isopropanol and spun again. The supernatant was removed and the DNA pellet was washed with 70% ethanol. The DNA was then dissolved in 100 µl of dH_2_O. One microlitre was used per PCR reaction. PCR was performed with the primers: AAGCCAAACAGCGGATAGCA and TGGTTGGCAGTAGGTCTTCG. PCR products were gel verified and sent for sequencing. The sequencing of genomic DNA from heterozygous flies yielded an overlay of the DNA sequence from both chromosomes. The mutant sequence was then manually called from the chromatogram.

The *vih^Null^* alleles have either 1- or 4-nucleotide deletion, resulting in a frame-shift at the 8th amino acid of the gene, which disrupts all domains of the protein. The *vih^Null^* alleles were created using different Cas9-expressing lines in different genetic backgrounds. None of these alleles are homozygous viable after larval stage 3, and they do not complement two *vih* deficiencies nor the *vih^KG02013^* allele [34]. We were able to rescue the viability of the two null alleles with ubiquitously expressed Vih^WT^ and Vih^Δ^^N-9^ but we were not able to rescue viability with Vih^Δ^^CS^, a catalytically dead version of Vih, and only the transgenically expressed Vih^WT^ allele was able to rescue the fertility (data not shown).

The *vih^ΔN-9^* mutation is a deletion of 9 amino acids in the 27-amino acid N-terminal region. The *vih^ΔN-9^* allele is homozygous viable and complements two *vih* deficiencies. The other N-terminal allele, *vih^ΔN^* allele (*vih^ΔN-4^*) is a deletion of only 4 amino acids. This allele was created using a different Cas9-expressing line directly on an FRT chromosome, and was, therefore, created in a completely different genetic background. Transheterozygous *vih^ΔN-9^*/*vih^ΔN-4^* has the same phenotype as the *vih^ΔN-4^* allele alone and neither alleles have a phenotype when crossed to a *vih* deficiency (data not shown). The phenotype that was present in both alleles increased with age and an age of 14 days was chosen for analysis, unless otherwise stated. The third chromosome with the *vih^ΔN-9^* allele on it was isogenized after the stock was originally created and rebalanced.

### 4.4. Western blots

For the western blots of *Drosophila* ovaries, 30 ovaries were dissected in 60 µl of PBS containing Protease and Phosphatase inhibitor (Roche). The samples were lysed using a homogenizing pestle (Sigma-Aldrich), and the lysates were clarified by centrifugation. Two times Laemmli buffer was added to the cleared lysates, and the samples were boiled 2 min. The proteins were then resolved by SDS–PAGE. The electrophoretic run was performed using a Mini-PROTEAN Tetra Cell System (BioRad), in a Running Buffer solution (25 mM Tris, 192 mM glycine and 0.1% SDS, pH approx. 8.6, Sigma), at 200 V. The gel was assembled in a ‘transfer sandwich’ (cushion pad–filter paper–gel–membrane–filter paper–cushion pad) and blotted on a nitrocellulose membrane (GE Healthcare) for 2 h at 60 V in Transfer Buffer solution (25 mM Tris, 190 mM Glycine, 20% Methanol). Protein transfer was verified by Ponceau S staining, and the membrane was incubated for 45 min at room temperature in a blocking solution containing 5% milk (Marvel) and 0.1% Tween (Sigma) in PBS. The membrane was then incubated in a primary antibody solution prepared in blocking solution for 1 h at room temperature. The membrane was washed three times for 10 min at room temperature with a solution of 0.1% Tween in PBS (PBST) and then incubated in a secondary antibody solution prepared in blocking solution for 1 h at room temperature. The membrane was washed three times with PBST for 10 min at room temperature, incubated with a peroxidase ECL substrate (Pierce), and the proteins were detected by exposing an X-ray film (Fuji). *Primary antibodies:* Mouse anti-α-tubulin (1 : 1000) Clone DM1A from Sigma, Mouse α-Polo (1 : 500) and Rabbit α-Vih (1 : 500), Glover Lab.

### 4.5. Western blots, destruction assays and ubiquitination assay intensity measurements

The band intensity was calculated using FIJI, a box was drawn over the band and decreased to only include to area of interest, the background was subtracted for each band and normalized to the loading control (α-tubulin). The relative intensity was calculated by dividing by the control.

### 4.6. Egg laying and hatching rate

In large cages, 50 virgin females were mated with equal numbers males and maintained on apple juice agar plates with fresh yeast; the cages were kept at 25°C. The number of eggs laid during 2 × 2 h collections were counted every 1–3 days for a 21- or 28-day period (*n* = 2). The number of flies in the cadges were kept equal throughout the course of the experiment, the number of female flies did not drop below 40. After the eggs were counted, the plates were kept at 25°C for 5 extra days and examined for the number of larvae hatched. The hatch rates were calculated by dividing the number of larvae that eclosed by the total number of eggs laid.

### 4.7. Stock maintenance, clone induction, ageing, dissection and immunofluorescence

*Fly sample preparation*: all females were maintained at 25°C, mated and fattened for 16–24 h with yeast prior to dissection. *vih^ΔN-9^* flies, including all allele combinations, were aged, alongside controls, for 14 days unless otherwise specified. *polo* flies were dissected within a week after eclosion. *Rab6^D23D^, FRT40A* flies were crossed to FRT40A GFP and vials with wondering larvae were heat shocked for 2 h at 37°C for three consecutive days and the adults were dissected after 6 days. *Ovary dissection:* ovaries were dissected in 0.2% PBT (PBS + 0.2% Tween), fixed for 20 min in 4% paraformaldehyde/PBT, washed with PBT, blocked with PBT + 10% BSA for 1 h, and incubated with the primary antibody in PBS + 2% Tween + 1% BSA for 16–24 h at 4°C. After washing the ovaries with PBT three times for a total of 25 min, they were incubated with the secondary antibody for 2 h at RT, or 16 h at 4°C. Phalloidin staining for visualizing F-actin was done for 20 min, either after fixing or after secondary antibody staining. Finally, the ovaries were washed twice with PBT for a total of 40 min and mounted in Vectashield (Vector) with DAPI for visualizing DNA. The microtubule sample preparation and staining was performed as previously published [65]. Unless specified, all steps were performed at room temperature. *Internal controls:* GFP containing flies were used and taken through the whole procedure (from fattening to imaging) mixed with the mutant samples to ensure that the membrane phenotype was not due to physical damage. *Primary antibodies:* mouse α-Orb 4H8 and 6H4 (1 : 100 each) from Developmental Studies Hybridoma Bank (DSHB), was a kind gift from Daniel St Johnston. Guinea Pig α-Asterless (1 : 40 000) was a kind gift from Nasser Rusan. Mouse anti-α-tubulin (1 : 100) Clone B-5-1-2 from Sigma. Mouse α-Dap NP1-s (1 : 10) from DSHB. Rabbit α-Cyclin E (1 : 100) from Santa Cruz Biotechnology. Mouse α-Hts 1B1-s (1 : 30) from DSHB. Mouse α-Polo (1 : 50), Rabbit α-Vihar (1 : 500), Mouse α-Dynein IC74 (1 : 1000) and Chicken D-Plp (1 : 1000), Glover Lab. *Secondary antibodies:* (all 1 : 500) Goat α-Chicken 488 from Life Technologies, Goat α-Mouse 488 and 647 from Life Technologies, Goat α-Guinea Pig 488 from Invitrogen and 647 from Life Technologies, Goat α-Rabbit 488 from Invitrogen and 647 from Life Technologies. *Small molecule stains:* Alexa Fluor™ 568 Phalloidin (1 : 200) from Thermo Fisher Scientific, CellMask Deep Red Plasma Membrane Stain (1 : 1000) from Thermo Fisher Scientific.

### 4.8. Intensity measurements of fixed samples

For the Plp and Dynein experiments, the egg chamber samples were prepared in parallel and treated the same. The control was analysed first to establish the confocal settings to be used, which remained unaltered for all images acquired, for the channel being directly compared (which was the 488 channel for all experiments). The intensity measurement was taken using FIJI measurement tool. The measurement section chosen for the Plp and Dynein was a circle drawn to the edges of a nucleus at early time points and used for all images as well as for S5/6, and the pixel histogram was checked to only include the signal of interest. Special care was taken to not include the centrosome-associated Plp or Dynein fluorescence in the intensity measurement analysis, since they are closely associated with the oocyte nucleus and germinal vesicles in the control but not *vih^ΔN-9^* egg chambers. Stacks of egg chambers were taken every 1 µm and the brightest single image was chosen for the intensity measurement analysis. The intensity was subtracted for the background in each individual image and the relative intensity was calculated by dividing by the average of the control.

### 4.9. Live sample preparation

After fattening, 14-day aged mated females that were maintained at 25°C for 16–24 h were dissected and their ovaries were short-term live imaged (1 h at room temperature) by dissecting directly in voltelef oil or in PBS with CellMask (incubated for 15 min prior to being transferred to voltelef oil for imaging).

### 4.10. Microtubule nucleation live-imaging sample preparation

Ovaries from flies aged for 14 days were dissected for *in vivo* preparation as previously described [66,67]. Images were acquired with a ZEISS LSM 880 Airyscan confocal microscope and analysed using ImageJ. *In vivo* images were obtained at approximately 25°C with a 40×/1.2 numerical aperture (NA) water immersion objective. Single-focal planes were taken every 2 s and processed with proprietary ZEISS' ZEN 2.1 software for Airyscan resolution optimization. We took an unbiased sampling of the centrosomes that satisfied the binary decision of in the oocyte or not. Additionally, the measurements were normalized for filming time with the centrosomes in focus: for the controls, the number of ectopic centrosomes analysed was 17 and the total duration of movies was 56.7 min. The total time of focused centrosomes was 42.6 min. At this time, the number of comets was 4. Therefore, the nucleation efficiency (how long it takes a two-week-old control centrosome to nucleate 1 microtubule) was 10.65 min. For the *vih^ΔN-9^* egg chambers, the number of ectopic centrosomes analysed was 19 and the total duration of movies was 63.3 min. The total time of focused centrosomes was 47 min. In this time, the number of comets was 2. Therefore, the nucleation efficiency was 23.5 min.

### 4.11. Ubiquitination assay

For the ubiquitination assay, *Drosophila* APC/C was immuneprecipitated from 2 g of pMTB-Apc4-TAP embryos. The samples were resuspended in 10 ml of ice-cold Buffer A (75 mM HEPES pH7.5, 150 mM KCl, 1.5 mM EGTA, 1.5 mM MgCl2 a, 7.5% glycerol, 0.1% NP40) containing fresh DTT (5 mM) and complete protease inhibitor cocktail (Roche). Embryos were broken using a glass homogenizer and the lysates were cleared by centrifugation at 20 000 rpm for 30 min. The cleared lysates were mixed with pre-equilibrated Dynabeads (Invitrogen) conjugated with rabbit IgG (MP Biochemicals) and incubated for 2–4 h with gentle rotation. Non-specific bound proteins were removed by six successive washes in Buffer A which contained low salt followed by a final wash with Buffer B (50 mM Tris–HCl pH 8.0, 0.5 mM EDTA). The E2 enzymes His-Vih^WT^, His-Vih^ΔN-9^, His-Vih^ΔCS^ and His-Ube2 s were expressed in *Escherichia coli* (strain Bl21 cod+) by incubating the cultures overnight at 18°C with 0.1 mM of IPTG. The fusion proteins were then purified using Ni-NTA Agarose beads (QIAGEN). Substrates were labelled with [35S] methionine (PerkinElmer) in a coupled *in vitro* transcription–translation (IVT) system (Promega). His-Fzy was purified from insect cells using the baculovirus-based expression system. Briefly, a plasmid containing His-Fzy was transformed into MAX Efficiency DH10Bac competent cells (Thermo Fisher Scientific) and the bacmids carrying His-Fzy genes were purified according to the manufacturer's instructions. The bacmids were handed to the Baculovirus Facility at the Department of Biochemistry of the University of Cambridge for insect cells transfection, virus production and titration. His-Fzy viruses were transduced into Sf9 cells at an MOI of 10. His-Fzy was purified from cell pellets from 800 ml of liquid culture of Sf9 cells using Ni-NTA agarose (QIAGEN), according to the manufacturer's instructions. Ubiquitination reactions were performed at 27°C in 10 µl of the buffer (20 mM Tris–HCl [pH 7.5], 100 mM KCl, 2.5 mM MgCl2) containing 4 µl of purified APC/C, 3.5 µM of E2 enzymes, 0.75 mg ml^−1^ ubiquitin, 1 µM ubiquitin-aldehyde, 200 µM MG132, 200 µM DTT, 2 mM ATP and 1 µl *in vitro*-translated (IVT) Mes1 substrate [68]. Reactions were stopped at the indicated time points with SDS sample buffer and mixtures were resolved by SDS–PAGE.

### 4.12. Destruction assay

The *in vitro* destruction assay in *Xenopus* egg extracts was performed as described before [69]. Briefly, ^35^S-methionine-labelled substrate proteins were prepared in a coupled *in vitro* transcription–translation system (Promega) according to the manufacturer's instructions. Cytoplasmic extracts of cytostatic factor (CSF)-arrested *Xenopus* eggs were prepared following standard procedures. The CSF extracts were first released into interphase by addition of 0.4 mM CaCl_2_ and 10 µg ml^−1^ cycloheximide, and then incubated for 2-4 h at 23°C. Substrates were added to the extracts and the reactions were started by adding 150 ng of purified *Xenopus* Fzr (gift from Hiro Yamano) to the mixture. Aliquots were collected into 2× Laemmli buffer at 0, 1, 2 and 3 h, boiled for 2 min and resolved by SDS–PAGE.

### 4.13. Colcemid drug treatment

The colcemid treatment was performed exactly as previously published [24].

### 4.14. Statistical test

*z*-test for proportions: the *n*-values are given on the figures and the *p*-values are given in the figure legends. Shapiro–Wilk for normalcy: all non-proportional datasets were tested for normalcy and all came back with skewed bell curve distributions (not normal). Wilcoxon signed-rank test for under 20 samples (given as a range because it is an estimate with small sample sizes): the *n*-values are given on the figures and the *p*-value ranges are given in the figure legends. Welch's unbiased two-tailed *t*-test used for non-normal sample sizes of 20 or greater: the *n*-values are given on the figures and the *p*-values are given in the figure legends. When this test was used the non-parametric Wilcoxon signed-rank test was also performed and the *p*-values obtained fell within the range given by Welch's unbiased two-tailed *t*-test. This was used to give a specific *p*-value when possible. For results with binary categorical variables (e.g. presence or absence of a phenotype), *p*-values were calculated by performing two-tailed Fisher's exact tests.

## Data accessibility

The data that support the findings of this study are available from the corresponding authors upon reasonable request. Correspondence and materials requests should be sent to A.L.B (alb84@cam.ac.uk) or Y.K. (yk299@cam.ac.uk). The data are provided in electronic supplementary material [70].
